# Upregulation of *abeM*, *amvA*, and *qacEΔ1* efflux pump genes associated with resistance of *Acinetobacter baumannii* strains to disinfectants

**DOI:** 10.1002/hsr2.395

**Published:** 2021-10-01

**Authors:** Tahereh Rostami, Mojtaba Ranjbar, Sedighe Ghourchian, Fatemeh Darzi, Masoumeh Douraghi, Mahmoud Nateghi‐Rostami

**Affiliations:** ^1^ Faculty of Biotechnology Amol University of Special Modern Technologies Amol Iran; ^2^ Department of Pathobiology School of Public Health, Tehran University of Medical Sciences Tehran Iran; ^3^ Department of Parasitology Pasteur Institute of Iran Tehran Iran

**Keywords:** *Acinetobacter baumannii*, biocides, disinfectants, efflux pumps

## Abstract

**Background and aims:**

*Acinetobacter baumannii* is among the most concerning cause of nosocomial infections due to its high level of antibiotic resistance and high mortality. The aim of this study was to determine the role of efflux pumps in resistance of *A. baumannii* strains to three disinfectants, including MICROZED ID‐MAX, NANOSIL D2, and OPIDEX OPA.

**Methods:**

Twenty‐eight environmental and clinical isolates of *A. baumannii* were collected from selected hospitals of central Iran. The minimum inhibitory concentrations of the disinfectants were determined and real time reverse transcriptase‐PCR was performed to investigate the expression level of *qacEΔ1*, *amvA*, *abeM*, and *adeB* efflux pump genes.

**Results:**

Considering both clinical and environmental isolates, there was a significant difference in the mean expression level of *qacEΔ1* gene between susceptible and resistant strains to MICROZED ID‐MAX disinfectant, of *amvA* and *abeM* genes between susceptible and resistant strains to NANOSIL D2 disinfectant and of *abeM* gene in susceptible and resistant strains to OPIDEX OPA disinfectant (all *P* ˂ .05). The expression levels of *abeM* and *amvA* genes were higher in the environmental isolates that were resistant to NANOSIL D2 disinfectant compared to those that were susceptible (*P* ˂ .05).

**Conclusions:**

This study provided evidence for the role of *abeM* and *amvA* genes in the resistance of environmental isolates to disinfectants, particularly hydrogen peroxide derivatives.

## 1 INTRODUCTION

Health‐care associated infections or nosocomial infections are the leading cause of mortality in hospitalized patients and are regarded as a concern for both patients and medical staff.^1^ These infections are usually caused by resistant microbial strains such as *Acinetobacter baumannii*, an emerging pathogen associated with various outbreaks in hospitals all over the world.^2^

*A. baumannii* are nonfermentative gram‐negative coccobacilli that colonize various organs of hospitalized patients and could survive for long time on both humid and dry environmental surfaces.^3^, ^4^ There are increasing reports of carbapenem‐resistant and multidrug resistant *A. baumannii* and several evidence show dissemination of common clones of drug resistant *A. baumannii* strains among patients within or between hospitals. *A. baumannii* can tolerate desiccation and survive in the inanimate healthcare environment including surfaces and equipment for long period of time. A combination of these properties and the enhanced resistance of *A. baumannii* to antibiotic and biocide, make it a great challenge in healthcare settings. *A. baumannii* causes pneumonia, bacteremia, secondary meningitis, burn and wound infection, urinary tract infection, and soft tissue infection.^5^, ^6^, ^7^

Disinfectants are chemicals that inactivate pathogenic microorganisms on contaminated equipment and surfaces by different mechanisms. Various commercially available disinfectants are currently used to reduce or completely eliminate the microbial burden of health care facilities^8^ and their activity depends on several factors such as temperature, concentration, chemical nature, and pH.^5^

One of the key mechanisms of low susceptibility/resistance of bacteria to biocides is the function of efflux transport systems. Efflux pumps are proteins which are localized in plasma *membrane* of bacteria^9^ and efflux function allows the microorganisms to regulate their internal environment by removing toxic substances, including antimicrobial agents, metabolites, and biocides. According to their substrates, composition, number of membrane spanning segments, and energy sources, bacterial efflux pumps are classified into seven families including the resistance‐nodulation‐cell division superfamily (RND), the small multidrug resistance family (SMR), the major facilitator superfamily (MFS), the ATP‐binding cassette superfamily (ABC), the multidrug and toxic compound extrusion protein family (MATE), the p‐aminobezoyl‐glutamate transporter, and the proteobacterial antimicrobial compound efflux.^10^, ^11^, ^12^

*A. baumannii* have been isolated from surfaces, equipment, solutions, monitors, and beds in hospitals and most of the *A. baumannii* infections are directly associated with the length of hospital stay, especially in the intensive care units. To control the emergence and outbreaks of multiple drug resistance (MDR) *A. baumannii* in health care settings, there is a need to focus simultaneously on environmental isolates in order to disrupt transmission of *A. baumannii* clones between environment and hospitalized patients and/or healthcare‐workers.^13^ In this study, we have analyzed the transcription levels of four efflux pumps genes from four different families including: *qacEΔ1* (SMR), *adeB* (RND), *amvA* (MFS), and *abeM* (MATE) in the environmental and clinical isolates of *A. baumannii*. The association of activity of efflux pumps with susceptibility/resistance to common commercially available disinfectants was also evaluated.

## 2 MATERIALS AND METHODS

### 2.1 Disinfectants

Three commonly known disinfectants, including MICROZED ID‐MAX (Atrineh Saziba, Iran), NANOSIL D2 (Kimiafaam Pharmaceutical, Iran), and OPIDEX OPA (Sung Kwang Pharm, Korea), which are routinely used in hospitals of Iran to disinfect surfaces, equipment, and medical devices, were selected. MICROZED ID‐MAX is a concentrated disinfectant solution classified as quaternary ammonium compounds. NANOSIL D2 is a ready to use disinfectant for surfaces and might be used in a wide range of ambient temperatures (0°C‐95°C) composed of hydrogen peroxide and silver ions. OPIDEX OPA is a ready to use solution composed of 0.55% ortho‐phthalaldehyde (Table 1).

**TABLE 1:** Properties of disinfectants

	Disinfectants		
	MICROZED ID‐MAX	NANOSIL D_2_	OPIDEX OPA
Composition	‐ Didecylmethylpoly(oxyethyl) ammonium propionate ‐ Polyethylene glycol ‐ *N*,*N*‐Dimethyldodecylamine ‐ Anti‐corrosion agent ‐ Complexing agent	Hydrogen peroxide and silver ion	Ortho‐phthalaldehyde 0.55 g/100 mL
Properties	‐ Yellowish clear liquid ‐ Medium disinfectant solution	‐ Ready to use disinfectant ‐ Usable in a wide range of thermal ranges (0°C‐95°C)	‐ Ready to use disinfectant ‐ Transparent light blue liquid pH: 7.5
Mechanism of action	‐ Inactivation of the cellular enzyme system and protein degradation and cell membrane disruption	‐ Binding of silver ions to cellular enzymes and inhibition of bacterial cell metabolic activities ‐ Damage to the cell wall by oxygen radicals released from hydrogen peroxide	‐ Protein degradation of microorganisms ‐ Prevent of spore germination

### 2.2 Settings and sampling

Samples were collected from environmental surfaces, equipment, and hospitalized patients of intensive care unit (ICU) and neonatal intensive care unit wards of four hospitals located at central Iran including Qom and the capital Tehran provinces. For environmental sampling, the sterile swab was moistened with sterile saline and rubbed by swab over a 10 cm square surface of environment. In the case of liquids, 1 mL of the solution was used as sample for culture.^14^

Clinical MDR strains were isolated from skin ulcers, trachea, and bronchoalveolar specimens of the hospitalized patients. Also, environmental strains were isolated from bed sheets, sinks, faucets, carrying tables, and medical devices such as suction tubes. The clonal distribution of the isolates was previously determined which belongs to nine different clones.^14^

### 2.3 Identification of *A. baumannii* strains

The swabs were directly cultured on blood agar medium and then were subcultured on MacConkey agar medium (Merck, Germany) and incubated at 37°C for 48 hours. The resulting single colonies were used further for culture and biochemical tests. MacConkey agar, OF (oxidative fermentative basal agar), TSI (triple sugar iron agar) (all from Merck, Germany), oxidase (Padtan Teb Co, Iran), and growth test at 44°C were used to identify *A. baumannii*.^14^ Nonfermentative Gram‐negative bacilli which identified as *A. baumannii* were further confirmed by PCR targeting *bla*_OXA‐51_ gene. For this purpose, the strains were cultured on trypticase soy agar (TSA) medium and incubated at 37°C for 18 to 24 hours. DNAs were extracted by alkaline lysis method,^15^ then PCR of the target gene was performed using specific primers (F: 5′‐TAATGCTTTGATCGGCCTTG‐3′ and R: 5′‐TGGATTGCACTTCATCTTGG‐3′). The reaction consisted of 15.5 μL of distilled water, 2 μL of 10× buffer, 0.6 μL of MgCl_2_ (50 mM), 0.4 μL of dNTPs, 0.5 μL of each primer, 0.1 μL of *Taq* DNA polymerase, and 0.5 μL of DNA. The temperature steps include initial denaturation at 95°C for 5 minutes, then 35 cycles of denaturation at 95°C for 45 seconds, annealing at 58°C for 45 seconds, extension at 72°C for 45 seconds, followed by final extension at 72°C for 10 minutes. The presence of *bla*_OXA‐51_ gene was confirmed by the visualization of 353 bp band on 1% agarose gel after electrophoresis. Confirmed isolates were kept at −20°C in trypticase soy broth (Merk, Germany) solution until further use.

### 2.4 MIC and MBC determination

Minimum inhibitory concentration (MIC) and minimum bactericidal concentration (MBC) of disinfectants were determined by microdilution broth method.

#### 2.4.1 Preparation of disinfectants

A 2‐fold serially diluted concentrations of disinfectants were prepared in triplicate with distilled water in 96‐well microtiter plates. The concentrations prepared for MICROZED ID‐MAX disinfectants were from 2.5% to 0.019%, for NANOSIL D2 disinfectant were from 5% to 0.039%, and for OPIDEX OPA were from 100% to 0.78%. Although these disinfectants are ready‐to‐use solutions, a broader range around the concentrations recommended by the manufacturers were used to determine the MICs. In other words, concentrations recommended by manufacturers, along with one or two concentrations higher or lower, were applied to determine MICs. Numerous experiments were performed to obtain the optimum concentrations.

The disinfectants were kept in the standard conditions recommended by the manufacturers and their stability was evaluated by testing the MIC against a standard strain, *A. baumannii* ATCC 19606, in each round of experiment. One hundred microliter of each of eight concentrations of disinfectants were pipetted into each well of microtiter plates.

#### 2.4.2 Preparation of microbial suspensions

A suspension of 0.5 McFarland was prepared for each of the isolates using normal saline, then each microbial suspension was diluted 1:100 in Mueller Hinton Broth (MHB) medium (Merck, Germany) and 100 μL of bacterial suspensions were added to each well of plates containing disinfectant. The positive control wells contained 100 μL of 1% microbial solution without disinfectant and the negative control wells contained 100 μL of MHB solution without microbial suspension. *A. baumannii* strain ATCC 19606 was used as the standard strain. The experiments were performed in triplicate. The microtiter plates were incubated at 37°C for 18 to 24 hours. MIC is defined as the lowest concentration of a disinfectant that inhibited the visible growth of the bacteria after overnight incubation, and MBC is defined as the lowest concentration of a disinfectant that prevented the growth of the bacteria. MIC_50_ (concentration of disinfectant in which 50% growth of strains are inhibited) and MIC_90_ (concentration of disinfectant in which 90% growth of strains are inhibited) were determined as criteria for determining the susceptibility of the strains to each of the disinfectants. The isolates which had MIC concentrations ≤MIC_50_ were considered susceptible and isolates with MIC concentrations ≥MIC_90_ were considered resistant to each disinfectant.^16^ Overall, the isolates that were resistant to at least one of the three disinfectants were considered resistant, and isolates that were susceptible to all three disinfectants were considered susceptible.

#### 2.4.3 Minimum bactericidal concentration

After MIC determination, the well containing MIC and the well after that were cultured on TSA medium and incubated at 37°C for 18 to 24 hours. Then, plates without colony growth with the lowest disinfectant concentration were designated as MBC.

### 2.5 Real time reverse transcriptase‐PCR

RNA extraction of the samples was performed using Trizol reagents (Sigma), according to the manufacturer's instruction. RNA was kept at −70°C until further use. Measurement of absorption at wavelengths of 260 and 280 nm was performed to determine the concentration and purity of extracted RNA.

cDNA synthesis was performed by reverse transcription using M‐MLV enzyme (Takara, Japan). Briefly, 10 to 100 ng of RNA was mixed with 1 μL random hexamer and 2.5 μL diethyl pyrocarbonate (DEPC) treated water and incubated at 70°C for 5 minutes. After chilling on ice, 4 μL 5× buffer, 1 μL dNTPs, 0.5 μL RNasin, and 1 μL M‐MLV (10 000 U) were added to the tube and incubated for 1 hour at 37°C and 5 minutes at 70°C and used as cDNA samples.^17^

For real time PCR, in a 25 μL reaction mixture 9.5 μL of distilled water, 0.5 μL of each primer pairs (Table 2), 12.5 μL SYBR Green (Takara, Japan), and 2 μL cDNA were prepared. The cycling program were set on Corbett machine (Corbett Rotorgene 6000, Qiagen, UK) as: initial denaturation at 95°C for 15 minutes, 40 cycles of replication including denaturation at 94°C for 30 seconds, annealing at 55°C for 30 seconds, and elongation at 72°C for 30 seconds. Target genes were *qacEΔ1* (SMR), *adeB* (RND), *amvA* (MFS), and *abeM* (MATE).

**TABLE 2:** Sequence of primers of efflux genes

Gene		Primernucleotide sequence (5′ → 3′)	Expected size (bp)	Reference
*adeB*	F	GAATAAGGCACCGCAACAAT	124	38
	R	TTTCGCAATCAGTTGTTCCA		
*amvA*	F	GCCGCTCAATTATTTTGCCA	137	32
	R	TTGCTGCGCCACTACAACTA		
*qacEΔ1*	F	ATCGCAATAGTTGGCGAAGT	226	30
	R	CAAGCTTTTGCCCATGAAGC		
*abeM*	F	AGGGACGTATTATGGCGAAA	165	39
	R	CTGCTGTGCTTAGACCAATTTTT		
*16S rRNA*	F	CAGCTCGTGTCGTGAGATGT	150	30
	R	CGTAAGGGCCATGATGACTT		

The housekeeping gene 16S rRNA was used as the internal standard for normalization. qPCR results were calculated based on Pfaffl method using *C*_*t*_ value in 2^−ΔΔ*Ct*^ formula.^17^ The relative expression of each gene was calculated as the normalized ratio of gene expression of a given strain to gene expression in reference strain. *A. baumannii* strain ATCC19606 was used as the standard strain.

### 2.6 Statistical analysis

Data were analyzed using SPSS software ver. 20.0 (SPSS Inc., Chicago, Illinois). Parameters such as mean and SD were calculated as descriptive results. Pearson correlation coefficient and nonparametric tests of Mann‐Whitney *U* test and Kruskal‐Wallis were used to compare between groups. The correlation intensity is graded as follows: from 0.1 to 0.29 = weak, from 0.3 to 0.49 = average, from 0.5 to 1 = strong. Significance level less than .05 was considered significant.

## 3 RESULTS

### 3.1 Bacterial strains

During the 6 months of sampling, a total of 122 samples including 88 environmental and 34 clinical were collected from ICUs of four referral hospitals in central Iran. Of them, 31 were identified as *Acinetobacter* spp. using phenotypic tests (including OF fermentation, oxidase, and growth at 44°C). A total of 28 strains, including 10 environmental isolates and 18 clinical isolates were definitively identified as *A. baumannii* by amplification of *bla*_OXA‐51_.

### 3.2 MICs of three disinfectants

**FIGURE 1:**
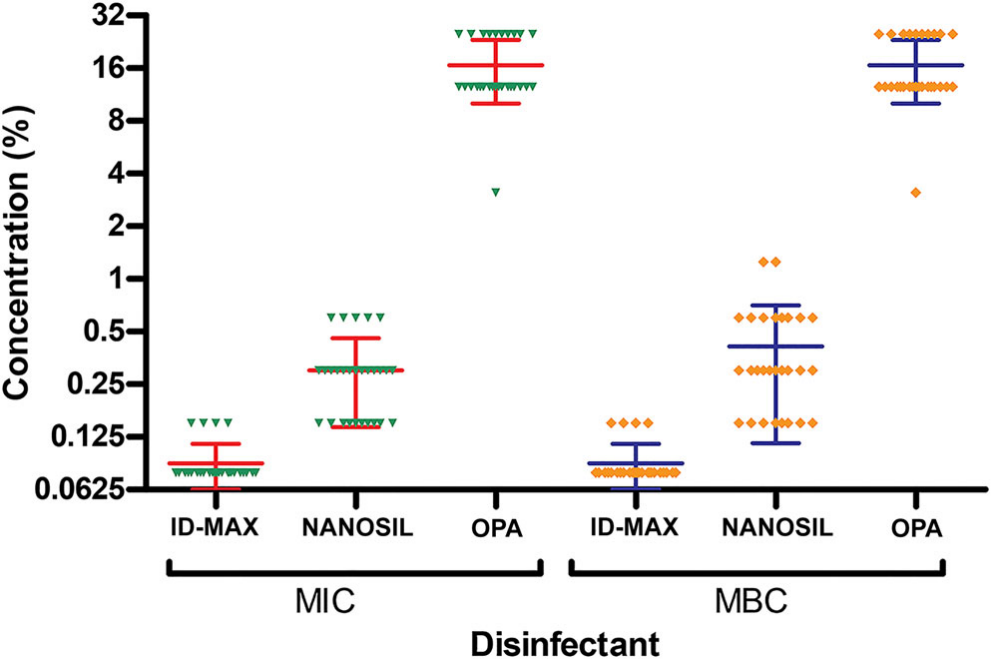
Minimum inhibitory concentration (MIC) and minimum bactericidal concentration (MBC) values of three disinfectants in *Acinetobacter baumannii* isolates. Mean of MIC and MBC values of three disinfectants has been shown in *A. baumannii* isolates. Mean and distribution of MICs and MBCs are shown. To determine MIC, different concentrations of disinfectants were applied to the microbial suspension by microdilution method. After MIC determination, part of their contents were cultured in trypticase soy agar medium 18 to 24 hours and wells without colony growth at the lowest concentration of disinfectant designated as MBC

MIC, MIC_50_, and MIC_90_ were determined for all three disinfectants and are shown in Table 3. Also, the mean of MICs and MBCs in all 28 strains was calculated and compared. The mean ± SD MIC of MICROZED ID‐MAX disinfectant was 0.09 ± 0.026, NANOSIL D2 was 0.3 ± 0.16, and OPIDEX OPA was 16.62 ± 6.6. The mean MBC of MICROZED ID‐MAX disinfectant was 0.09 ± 0.026, NANOSIL D2 was 0.41 ± 0.29, and OPIDEX OPA was 16.62 ± 6.6 (Figure 1). Based on the Mann‐Whitney *U* test, the difference between the mean MICs of NANOSIL and other disinfectants (*P* < .05), and between OPA and other disinfectants (*P* < .001) were significant. Also, the difference between the mean MBCs of NANOSIL and other disinfectants (*P* < .05), and between OPA and other disinfectants (*P* < .05) were significant. Then, susceptible and resistant strains were determined based on MIC_50_ and MIC_90_. The MIC_50_ and MIC_90_ in MICROZED ID‐MAX disinfectant were 0.07% and 0.15%, in NANOSIL were 0.31% and 0.62%, and in OPIDEX OPA were 12.5% and 25%, respectively. According to MIC_50_ and MIC_90_, the highest percentage of strains was susceptible to MICROZED ID‐MAX disinfectant (24 strains, 86%), while NANOSIL D2 (23 strains, 82%) and OPIDEX OPA (18 strains, 64%) disinfectants ranked second and third, respectively. Therefore, the strains had the highest resistance to OPIDEX OPA disinfectant (Table 4).

**TABLE 3:** Serial dilutions of disinfectants for MIC determination

		Dilution number									
Disinfectants		1	2	3	4	5	6	7	8	MIC_50_ (%)	MIC_90_ (%)
MICROZED ID‐MAX	Serial concentrations of disinfectants (%)	2.5	1.25	0.62	0.31	0.15	0.07	0.03	0.01	0.07	0.15
	No. of strains containing specified MICs	0	0	0	0	4	24	0	0		
NANOSIL D2	Serial concentrations of disinfectants (%)	5	2.5	1.25	0.62	0.31	0.15	0.07	0.03	0.31	0.62
	No. of strains containing specified MICs	0	0	0	5	13	10	0	0		
OPIDEX OPA	Serial concentrations of disinfectants (%)	100	50	25	12.5	6.25	3.12	1.56	0.78	12.5	25
	No. of strains containing specified MICs	0	0	10	17	0	1	0	0		

Abbreviation: MIC, minimum inhibitory concentration.

**TABLE 4:** Susceptibility ranges of *Acinetobacter baumannii* isolates to three disinfectants

		Disinfectants									
		MICROZED ID‐MAX			NANOSIL D2			OPIDEX OPA			
Strains		MIC (%)	MBC (%)	Susceptible/resistant	MIC (%)	MBC (%)	Susceptible/resistant	MIC (%)	MBC (%)	Susceptible/resistant	Overall sensitivity
1	Clinical	0.078	0.078	Susceptible	0.15	0.6	Susceptible	12.5	12.5	Susceptible	Susceptible
2		0.078	0.078	Susceptible	0.3	0.6	Susceptible	12.5	12.5	Susceptible	Susceptible
3		0.078	0.078	Susceptible	0.15	0.15	Susceptible	12.5	12.5	Susceptible	Susceptible
4		0.078	0.078	Susceptible	0.3	0.3	Susceptible	12.5	12.5	Susceptible	Susceptible
5		0.15	0.15	Resistant	0.15	0.15	Susceptible	25	25	Resistant	Resistant
6		0.078	0.078	Susceptible	0.3	0.3	Susceptible	25	25	Resistant	Resistant
7		0.078	0.078	Susceptible	0.6	1.25	Resistant	12.5	12.5	Susceptible	Resistant
8		0.078	0.078	Susceptible	0.6	0.6	Resistant	12.5	12.5	Susceptible	Resistant
9		0.078	0.078	Susceptible	0.3	0.3	Susceptible	25	25	Resistant	Resistant
10		0.078	0.078	Susceptible	0.3	0.3	Susceptible	12.5	12.5	Susceptible	Susceptible
11		0.078	0.078	Susceptible	0.15	0.15	Susceptible	25	25	Resistant	Resistant
12		0.078	0.078	Susceptible	0.3	0.6	Susceptible	25	25	Resistant	Resistant
13		0.078	0.078	Susceptible	0.3	0.3	Susceptible	12.5	12.5	Susceptible	Susceptible
14		0.078	0.078	Susceptible	0.15	0.15	Susceptible	12.5	12.5	Susceptible	Susceptible
15		0.078	0.078	Susceptible	0.15	0.15	Susceptible	12.5	12.5	Susceptible	Susceptible
16		0.078	0.078	Susceptible	0.3	0.6	Susceptible	12.5	12.5	Susceptible	Susceptible
17		0.078	0.078	Susceptible	0.3	0.3	Susceptible	25	25	Resistant	Resistant
18		0.078	0.078	susceptible	0.6	0.6	Resistant	3.12	3.12	Susceptible	Resistant
19	Environmental	0.078	0.078	Susceptible	0.3	0.3	Susceptible	25	25	Resistant	Resistant
20		0.078	0.078	Susceptible	0.6	1.25	Resistant	25	25	Resistant	Resistant
21		0.15	0.15	Resistant	0.15	0.3	susceptible	25	25	Resistant	Resistant
22		0.078	0.078	Susceptible	0.3	0.3	Susceptible	12.5	12.5	Susceptible	Susceptible
23		0.078	0.078	Susceptible	0.15	0.15	Susceptible	12.5	12.5	Susceptible	Susceptible
24		0.078	0.078	Susceptible	0.3	0.6	Susceptible	12.5	12.5	Susceptible	Susceptible
25		0.15	0.15	Resistant	0.15	0.15	Susceptible	12.5	12.5	Susceptible	Resistant
26		0.15	0.15	Resistant	0.3	0.3	Susceptible	25	25	Resistant	Resistant
27		0.078	0.078	Susceptible	0.15	0.15	Susceptible	12.5	12.5	Susceptible	Susceptible
28		0.078	0.078	Susceptible	0.6	0.6	Resistant	12.5	12.5	Susceptible	Resistant

Abbreviations: MBC, minimum bactericidal concentration (the lowest antibacterial concentration that leads to bacterial death); MIC, minimum inhibitory concentration (the lowest concentration of antibacterial agent that prevents visible bacterial growth).

### 3.3 The overall frequency of susceptible/resistant strains among clinical and environmental isolates of *A. baumannii*

**FIGURE 2:**
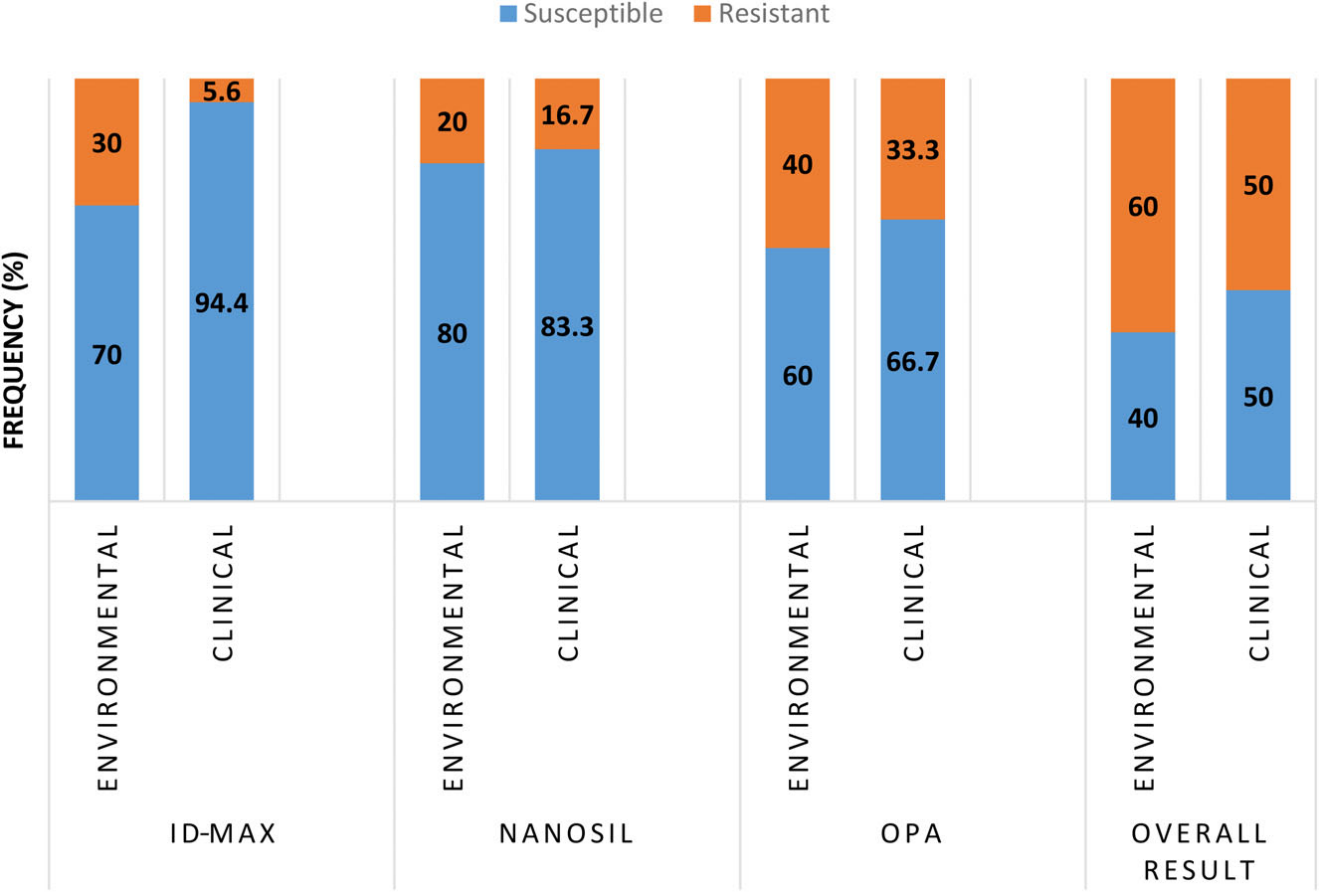
The frequency of susceptible and resistant strains of *Acinetobacter baumannii* to each disinfectant, in terms of environmental/clinical sources. Resistance of bacterial isolates to different disinfectants was calculated based on the minimum inhibitory concentrations. Overall, 60% of environmental and 50% of clinical isolates were designated as resistant strain

The frequency of resistant/susceptible strains to different disinfectants among environmental and clinical strains is shown in Figure 2. Of the total 28 strains, 10 were environmental (35.7%), of which 14.3% were susceptible and 21.4% were resistant. Also, 18 strains (64.3%) were clinical, of which 9 strains (32.1%) were susceptible and the same numbers were resistant. Thus, out of the total isolates, 13 strains (46.4%) were susceptible and 15 strains (53.6%) were resistant. According to these data, the frequency of resistance to all three disinfectants in environmental strains (n *=* 6 of 10) was significantly higher than that of clinical strains (n *=* 9 of 18) (*P* < .05) (Figure 2).

### 3.4 Distribution of efflux genes expression in susceptible and resistant strains

**FIGURE 3:**
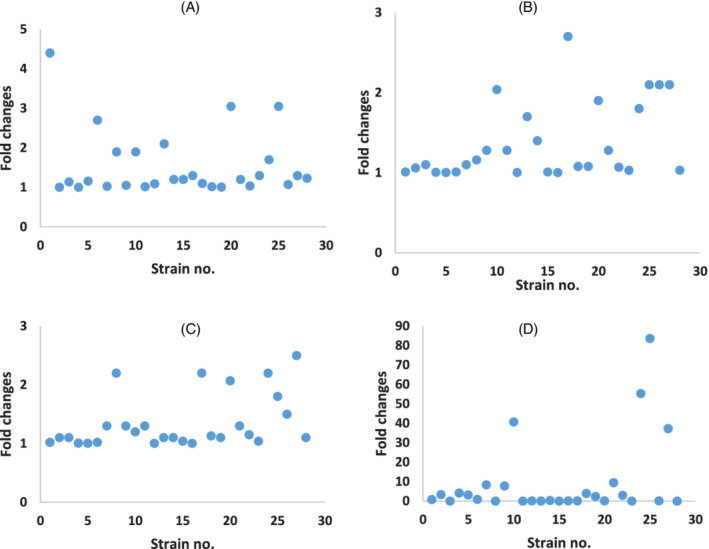
(A‐D). Relative expression of efflux genes in *Acinetobacter baumannii* strains isolated from clinical and environmental settings. Total RNA was extracted from each strain and reverse transcription to cDNA was performed using M‐MLV enzyme. Real‐time PCR was set on cDNA samples using SYBR Green I system and specific primer pairs. Threshold cycles (*C*_*t*_s) of each amplicon was used for further analysis. The relative quantities of the target genes were normalized against the 16 seconds rRNA gene. Fold‐expression changes of (A) *qacEΔ1*, (B) *adeB*, (C) *amvA*, (D) *abeM* genes were calculated in each strain compared to reference strain and represented

When comparing all strains in general (both environmental and clinical isolates), there was a significant difference in the mean expression level of *qacEΔ1* and *abeM* genes between susceptible (*qacEΔ1 =* 1.19 ± 0.08; *abeM = 7.0 ± 14.91*) and resistant (*qacEΔ1 =* 2.12 ± 2.07; *abeM = 24.04 ± 39.89*) strains to MICROZED ID‐MAX disinfectant (*P* ˂ .05). Also, there was a significant difference in the mean expression level of *amvA* (*P* < .05) and *abeM* (*P* ˂ .05) genes between susceptible (*amvA* = 1.57 ± 0.14; *abeM =* 2.45 ± 3.67) and resistant (*amvA* = 3.16 ± 2.09; *abeM* = 10.95 ± 21.88) strains to NANOSIL D2 disinfectant. Similarly, there was a significant difference in the mean expression level of *abeM* gene in susceptible (2.36 ± 3.45) and resistant (13.36 ± 24.25) strains to OPIDEX OPA disinfectant (*P* ˂ .05). The mean expression levels of *abeM* and *amvA* genes were significantly higher in environmental isolates which were susceptible (*amvA* = 1.82 ± 0.37; *abeM* = 3.02 ± 2.14) compared to those that were resistant (*amvA* = 4.09 ± 2.02; *abeM* = 23.86 ± 31.59) to NANOSIL D2 disinfectant (*P* ˂ .05). However, there was no significant difference between the mean expression of *abeM*, *qacEΔ1*, *amvA*, and *adeB* genes in resistance vs susceptible clinical isolates (Figure 3).

### 3.5 The correlation between the rates of expression of the studied genes

The correlation between the expression of four studied genes was investigated, and the results showed that the relationship between gene expression of *amvA* and *adeB* was positive, strong, and significant (*P* < .001), between *abeM* and *adeB* (*P* < .05) and between *abeM* and *amvA* (*P* < .05) was also significant.

## 4 DISCUSSION

In this study, the resistance of *A. baumannii* isolates to disinfectants (MICROZED ID‐MAX, NANOSIL D2, and OPIDEX OPA) was measured based on MIC. Then, the association between efflux gene expression rate and MIC changes was determined. The serial dilutions of the three disinfectants used in this study were prepared considering the recommended concentrations of the manufacturers. However, the recommended concentration, which is also applied in hospitals, was higher than enough to inhibit the growth and the isolates were inhibited or killed with lower dilutions. The maximum concentration of OPIDEX OPA disinfectant that inhabited the growth was a solution of 25% while the manufacturer recommended the concentration of 100%, and for NANOSIL D2 it was 0.6% while the recommended concentration was 100%. Similarly, in several other studies, the MIC of the biocides used was less than that of recommended by manufacturers.^18^, ^19^ For instance, in the study of the effect of hospital disinfectants including quaternary ammonium compounds (similar to MICROZED ID‐MAX) on *Staphylococcus epidermidis*, it was suggested that the MIC was from 6 to 8 times lower than the concentrations recommended by the manufacturer.^20^ However, there are studies that reported that all *A. baumannii* isolates were resistant to recommended concentrations of disinfectants.^21^

The use of concentrations above the effective level may lead to the emergence and spread of more resistant strains due to selective pressure and becomes a great challenge in nosocomial infection control.^19^, ^21^, ^22^, ^23^, ^24^ It was noted that the high concentrations of biocides are toxic to humans and the environment, in addition to imposing cost to health system.^22^ It seems that the difference in the effective concentration of disinfectants depends on the type of disinfectant or the bacteria tested.^2^, ^16^, ^25^ However, it can be assumed that the concentrations recommended by the manufacturers probably target all common infectious agents, not just a particular species such as *A. baumannii*.

In this study, MIC and MBC of MICROZED ID‐MAX disinfectant were equal in all isolates. Similarly in the OPIDEX OPA disinfectant, MIC and MBC were the same for all isolates. This point to the concentrations of these disinfectants applied here, which do not inhibit the growth of the isolates but kill them. In another study on two species of *Enterococcus*, didecyldimethyl ammonium chloride disinfectant had a similar pattern as its MIC and MBC were the same against the tested strains; *Enterococcus faecalis* and *Enterococcus faecium*.^26^

According to the results, the rate of resistance to OPIDEX OPA was significantly higher than that of the other two disinfectants. The high resistance of *A. baumannii* to OPIDEX OPA disinfectant may justify the use of high concentrations of this disinfectant in hospitals, according to the manufacturer's recommendation. It should be noted that the variation in response of *A. baumannii*'s to these three disinfectants is not unexpected owing to the difference in structure and mechanisms of disinfectant tested here. In another study, a significant association between the MICs of benzalkonium chloride and benzetonium chloride was attributed to the similarity of their chemical structure.^16^

According to this study, the percentage of resistant strains in environmental isolates (60%) was higher than that of resistant strains in clinical isolates (50%). This may be due to the fact that environmental strains are more exposed to disinfectants than clinical strains. In contrast to the current study, a study showed that clinical strains of *A. baumannii* were more resistant to disinfectants than environmental strains.^27^ The reason for the difference can be related to different testing conditions such as geographical location, type of disinfectants, and to the method of using disinfectants. The effectiveness of a disinfection method depends on the contact time, temperature, concentration of the active substance and the persistence of disinfectants,^2^ and the type of growth of the isolates in the environment (biofilm or plankton).^28^

In this study, the level of efflux gene expression was compared in environmental and clinical strains and showed that the mean expression of *amvA* and *adeB* genes was significantly higher in the environmental than clinical isolates of *A. baumannii*. There is further evidence for the role of *adeB* and *amvA* gene expression in resistance to biocides. In one study, strains of *A. baumannii* exposed to chlorhexidine digluconate had increased *adeB* gene expression.^29^ Also, the expression of the *adeB* gene in *A. baumannii*, which was resistant to triclosan, was significantly higher than the susceptible strains.^30^, ^31^ In MDR *A. baumannii*, which were exposed to methyl viologen and ethidium bromide, *amvA* expression levels increased compared to the susceptible strains and inhibiting the *amvA* pump confirmed the role of this gene in resistance.^32^ The most probable reason for the high resistance of environmental isolates is that they are more exposed to biocides than clinical isolates.

Based on the current result, the difference in the expression of *qacEΔ1* gene was significant between susceptible and resistant strains to MICROZED ID‐MAX disinfectant. Previously, a relationship between the presence of *qac* gene and increased MIC in *A. baumannii* isolates was reported.^33^ MICs were suggested to be significantly high in strains of *Salmonella* spp., *Escherichia coli*, *Klebsiella pneumoniae*, and *Staphylococcus aureus* containing the *qacEΔ1* gene.^25^, ^34^ In contrast, there are other studies that do not suggest a significant difference between the presence or absence of *qacE* gene and susceptible/resistant strains of *A. baumannii*.^18^, ^23^

This study showed that among clinical isolates, there was no significant difference in *amvA* gene expression between susceptible and resistant ones. There was also no significant difference in *abeM* gene expression between susceptible and resistant clinical isolates. In contrast, there was a significant difference in efflux gene expression between resistant and susceptible isolates of environmental source.

A study by Rajamohan et al suggested that by inactivation of these genes, susceptibility to multiple antibiotics and disinfectants was increased.^32^ While we did not find a role for *adeB* efflux gene function in disinfectant resistance, in other studies the role of efflux genes including the *adeB* gene in antiseptic‐resistant isolates of *Acinetobacter* spp. was demonstrated.^35^, ^36^

Based on the current results, there was a positive significant correlation between gene expression of *abeM* and *amvA*, *abeM* and *adeB*, and *amvA* and *adeB* in all samples, both clinical and environmental, and resistant and susceptible. This significant association among the expressions of efflux genes indicates that efflux's mechanism of biocides occurs through multiple genes function.

The results showed that the effective concentrations of disinfectants in inhibiting the growth of *A. baumannii* strains are less than the amounts recommended by manufacturing companies. Therefore, to prevent selective pressure and consequently the spread of biocide‐resistant strains, it is advisable to keep the concentration of the disinfectants according to their calculated MICs and bacterial type. EU GMP and US FDA recommend the use of disinfectants by rotations to prevent microbial resistance.^37^

## 5 CONCLUSIONS

The significant difference in the expression of efflux pumps genes studied in this study between the susceptible and resistant strains of *A. baumannii* shows the association of efflux function in resistance to disinfectants. Particularly *abeM* and *amvA* genes contribute to resistance of environmental isolates of *A. baumannii* to hydrogen peroxide derivatives. However, the contribution of efflux pumps mechanism in reducing the susceptibility of the clinical strains to disinfectants was not as evident as in the environmental isolates.

## FUNDING

This study was funded by Tehran University of Medical Sciences, Tehran, Iran. Tehran University of Medical Sciences had no role in the study design; collection, analysis, and interpretation of data; writing of the report; and the decision to submit the report for publication.

## CONFLICT OF INTEREST

The authors declare that they have no conflict of interest.

## AUTHOR CONTRIBUTIONS

Conceptualization: Mahmoud Nateghi Rostami

Data Curation: Mojtaba Ranjbar

Formal Analysis: Tahereh Rostami, Mahmoud Nateghi Rostami

Methodology: Tahereh Rostami, Mojtaba Ranjbar, Sedighe Ghourchian, Fatemeh Darzi

Supervision: Masoumeh Douraghi

Writing—Original Draft Preparation: Tahereh Rostami

Writing—Review and Editing: Masoumeh Douraghi, Mahmoud Nateghi Rostami

All authors have read and approved the final version of the manuscript.

Mahmoud Nateghi Rostami had full access to all of the data in this study and takes complete responsibility for the integrity of the data and the accuracy of the data analysis.

## TRANSPARENCY STATEMENT

Mahmoud Nateghi Rostami affirms that this manuscript is an honest, accurate, and transparent account of the study being reported; that no important aspects of the study have been omitted; and that any discrepancies from the study as planned (and, if relevant, registered) have been explained.

## Data Availability

The authors confirm that the data supporting the findings of this study are available within the article.
